# Interpretive ethnographic studies and in-depth qualitative research should be treated as high-quality evidence in the science of team science

**DOI:** 10.3389/fpsyg.2026.1781383

**Published:** 2026-06-03

**Authors:** Stephen Molldrem, Kevin Wooten

**Affiliations:** 1 Institute for Bioethics and Health Humanities, The University of Texas Medical Branch, Galveston, TX, United States; 2 Institute for Translational Sciences, The University of Texas Medical Branch, Galveston, TX, United States; 3 Department of Management, The University of Houston, Clear Lake, Houston, TX, United States

**Keywords:** evidence, qualitative research, science and technology studies, science of team science, team science

## Abstract

The Science of Team Science (SciTS) has an evidence problem. On the one hand, there are few studies demonstrating the effectiveness of evidence-informed team science trainings. On the other hand, SciTS has taken a relatively narrow view of what constitutes high-quality evidence of effectiveness. The field tends to privilege evidence generated from randomized controlled trials (RCTs), surveys of expert opinion, systematic reviews, and other methods of evaluation and assessment that are premised on narrow, experimental, or positivist understandings of what forms of knowledge are permitted to count as high-quality evidence. In this commentary, we suggest that SciTS ought to expand its understanding of what forms of knowledge can count as high-quality evidence. We argue that SciTS should include in-depth qualitative and ethnographic research programs that are not oriented around reproducibility or generalizability but instead focus on the value of specific studies, interpretive methods, and contextualized knowledge.

## Introduction

The Science of Team Science (SciTS) has an evidence problem. On the one hand, there are few studies demonstrating the effectiveness of evidence-informed team science trainings (Rolland et al., 2021a). On the other hand, SciTS has taken a relatively narrow view of what constitutes high-quality evidence of effectiveness. The field tends to privilege evidence generated from randomized controlled trials (RCTs), surveys of expert opinion, systematic reviews, and other methods of evaluation and assessment that are premised on narrow, experimental, or positivist understandings of what forms of knowledge are permitted to count as high-quality evidence (National Academies of Sciences, Engineering and Medicine, 2015). Favored approaches tend to valorize standards of rigor that lead to knowledge about how to effectively train science teams that can be characterized as “generalizable.” However, prioritizing approaches that rely on such standards precludes taking a more expansive view of what can be learned from in-depth qualitative studies of teams' successes or failures conducted in constructivist or interpretivist traditions. These fields use different standards

of rigor and do not aim to generate generalizable knowledge in the positivist vein (Martinez Tyson et al., 2025); however, they produce valuable evidence that can be the source of novel best practices in team science (Kotarba et al., 2023). In this commentary, we sugggest that SciTS ought to expand its understanding of what forms of knowledge can count as high-quality evidence. We argue that SciTS should include in-depth qualitative and ethnographic research programs that are not oriented around reproducibility or generalizability but instead focus on the value of specific studies, interpretive methods, and contextualized knowledge.

The current paradigm for understanding evidence in SciTS emerges from a fusion of standards from psychology and hierarchies promulgated by the evidence-based medicine (EBM) movement. While useful in many contexts, these standards of evidence are often not particularly well-suited to generating knowledge about the functionality of science teams, which are highly complex units of analysis. Being rooted in the hierarchies of evidence required of EBM and allied areas of science, the current approach to evidence in SciTS excludes many forms of rigorous in-depth qualitative research from being treated as sources of high-quality evidence. This is because in EBM and related frameworks, qualitative knowledge is often presented at the bottom of hierarchies (Bluhm, 2016; Evans, 2003). However, this is not the case across all fields. For example, fields such as Science and Technology Studies (STS), the sociology and anthropology of science and technology, and human-centered design all have rigorous frameworks for studying and influencing science teams using in-depth qualitative approaches outside of EBM hierarchies (Dourish, 2006; Felt, 2017; Rhodes and Lancaster, 2019; Ribes, 2019; Ribes and Baker, 2007).

Considering the availability of interpretive methodologies, along with persistent challenges in generating high-quality evidence for team science interventions and science teams more broadly, we argue that SciTS ought to expand its understanding of what forms of knowledge get to count as high-quality evidence for understanding and improving science teams. We certainly recognize the value of RCTs in evaluating the efficacy of team science trainings as well as other forms of evaluation such as pre-/post- assessments, surveys, expert consensus reports, and systematic reviews. However, our experience also suggests that well-executed long-term ethnographic studies and other in-depth qualitative and interpretive methods should be treated as sources of high-quality evidence in SciTS, and that this research should be funded and prioritized.

Our position is rooted partly in the fact that science teams are incredibly complex units of analysis that often require deeply contextualized inquiry (Latour, 1987; Wooten et al., 2014). Their work takes place over long periods, can involve multiple grant applications, different overlapping projects, several institutions, non-collocated collaborations, mixed track records of successes and failures that take years to unfold, and a wide range of other factors that elude quantitative measurement or standards of generalizability favored by positivist epistemologies (Latour and Woolgar, 1979). The complexities faced by science teams therefore make controlled experiments, standard evaluation studies, or translating evidence generated from studying other team formations less ideal than in-depth observations, ethnographic approaches, and long-term qualitative studies undertaken with the interpretive aim of understanding what makes high-functioning teams work in their contexts.

After reviewing the landscape of what currently counts as high-quality evidence in SciTS, we describe how in-depth qualitative methods can be incorporated into the field as “evidence-making” practices (see, Rhodes and Lancaster, 2019). We then articulate how rigorously executed interpretive studies can generate high-quality evidence to support and strengthen science teams. We conclude by suggesting paths forward.

## What currently counts as high-quality evidence in SciTS

There is considerable literature involving what constitutes scientific evidence; however, it reflects a disciplinary focus from the physical sciences and positivist varieties of social and behavioral science (Chang, 2017; Goodman and Royall, 1988; Taper and Lele, 2004). Although the practice of team science is considered interdisciplinary, the closest disciplinary alignment involves the social and behavioral domains (Fiore, 2008; Hall et al., 2008; Stokols et al., 2008). Following the framework developed by the National Science Foundation (NSF)'s report on robust and responsible social and behavioral science, determination of evidence involves whether or not a specific finding can be independently verified (Bollen et al., 2015). Additionally, the report finds that robust scientific findings are those that are reproducible, replicable, and generalizable. Given these standards and given the stage of maturity of SciTS as a discipline, SciTS has a considerable amount of work ahead on the positivist front. However, notably, that understanding of rigor excludes interpretive approaches where reproducibility is not a goal, such as those favored by anthropology and other fields which focus on deep context rather than generalizability. By expanding the view of evidence to include these areas, SciTS can enhance its understanding of both evidence and rigor.

SciTS is only several decades old (Hall et al., 2018). Early founders clearly noted that for the field to evolve, it must develop and implement evidence-based strategies (Börner et al., 2010; Falk-Krzesinski et al., 2010). Research agendas were articulated at the onset, and indicated that multiple levels of research were needed, with foundational areas such as definitions and measurement techniques as most critical, along with a focus upon team dynamics, team structures, and contextual supports (Falk-Krzesinski et al., 2011). While progress has been made within the last decade, the most recent comprehensive review notes that a need for evidence-based research still exists (Hall et al., 2018). This is also inferred by other reviews and critiques (Bozeman and Boardman, 2014; Hall et al., 2019; Lerner et al., 2022; Little et al., 2017).

The evidence issue in team science partially stems from whether scientific teams are fundamentally different than general task teams. The question here involves whether scientific teams should be studied from the same theoretical framework and methodologies as have been used to study other groups and teams. Another issue involves the applied nature of SciTS. Using criteria developed to differentiate pure and applied science, SciTS is clearly an applied science, with the aim of improving science teams (Kotarba, 2014; Morrison, 2006; Shaw, 2022; Yaghmaie, 2017). The focus of most articles has been optimizing collaboration processes at the team or research center level of analysis, using many disciplines to solve complex research or social problems, and understanding how science is best conducted in a team-based structure. Thus, given the high degree of interdisciplinarity in SciTS, the field's stage of maturity, along with its applied nature, it is understandable why the field has had trouble in producing research that is reproducible, replicable, and generalizable. Expanding the view of evidence to include interpretive ways of knowing and in-depth qualitative studies is one way to expand the field's priorities and focus.

The most current review of the literature surfaced numerous important findings (Hall et al., 2018). First, more than 75% of the articles reviewed used archival data (publications, administrative data, etc.). Most importantly, however, was that no more than 10% of the studies reported used qualitative methods. Many reported studies found differential effects of gender, prior experience, extent of disciplinarity, etc. Other reported studies followed the empirical input—process—outcomes framework of variables used to study general task teams being applied to science teams (Kozlowski, 2018; LePine et al., 2008; Mathieu et al., 2008, 2019). One research area that has been attempting to establish evidence involves attempts to identify team science competencies (Bisbey et al., 2021; Brasier et al., 2023; Hall et al., 2019; Lotrecchiano et al., 2021; McGuier et al., 2025; Mendell et al., 2024). These studies have relied primarily on the use of qualitative methods involving subject matter experts attempting to identify relevant competencies from existing literature and through thematic analysis and decision-making techniques. While some were closer to an interpretive epistemic ethos, the methods used across these studies still generally sought to ascertain generalizability.

Recently, concerns have been raised concerning rigor and reproducibility in team science. Rolland et al. (2021b) detailed an implementation study that involved collaboration planning that was both scalable and reproducible (Rolland et al., 2021b). Rolland et al. (2021a) have also addressed these issues and suggested that the use of best practices in team science could be operationalized to generate reproducible studies. Frameworks and methodologies such as these are relatively rare in SciTS, and it is hoped that future studies will adopt similar approaches.

Recently, a robust RCT was conducted to study a team science intervention (Hawk et al., 2024). This study investigated a training intervention to produce collaboration readiness among early career scientists. While there were reported ceiling effects present, this design is perhaps the closest example of high-quality evidence in the positivist vein thus far in the team science literature. Unfortunately, the issue of replication often involves obtaining a large enough sample and use of standardized intervention materials to determine programmatic effects. Another recent example of a robust design involves a cluster-randomized implementation trial of team training (McGuier et al., 2025). Of particular importance is the fact that this was a mixed methods approach using both surveys and interviews. Interestingly, the Hawk et al. (2024) study documented many lessons learned from the use of controlled randomized design that limited the study's generalizability. The study by McGuier et al. (2025) showed significant gains in teamwork knowledge, but no significant effects upon skill use or work-related outcomes.

Another area of evidence comes in the form of evaluating and comparing programmatic team science efforts at the institute level (Hall et al., 2012; Vogel et al., 2014, 2021). Illustrative of this approach is Vogel et al. (2021), who examined cross-disciplinary team science efforts funded by the National Center for Advancing Translational Sciences. A system level framework of policies, procedures, processes, and structures was investigated. Team processes such as fluid team assembly, specialized project management, cross-agenda partnerships, and decision making based on clear scientific criteria and milestones were also deployed. Examination of 175 funded team projects illustrated various impacts upon publications and commercialization criteria.

It is important to incorporate the observations of the two National Academies' comprehensive reviews and recommendations relative to the current status of team science evidence (National Academies of Sciences, Engineering, and Medicine (ed)., 2025). The 2025 report provided clear strategies on not only what needed to be studied, but methodological approaches as well. As the report notes, “areas of prioritization may include but are not limited to studies that incorporate both qualitative and quantitative data to build empirical evidence about the science context and research evaluating institutional policies and supports for science teams” (National Academies of Sciences, Engineering, and Medicine (ed)., 2025, p. 192). The report also reviewed the successes over the previous decade and articulated a set of new challenges to be addressed by team science researchers (National Academies of Sciences, Engineering, and Medicine (ed)., 2025). Among these challenges were: (1) the complexity of competing dynamics, (2) multi-level interventions, (3) availability of subjects, (4) skepticism of social science methodologies (particularly qualitative methods), and (5) the lack of teaching of team science competencies. There are thus acknowledged opportunities for expanding the scope of qualitative and interpretive approaches across SciTS undertakings.

To date, SciTS has shown incremental gains in maturity. As shown in Table 1, we suggest four domains of evidence appear to have been reported thus far. Given the challenges noted by the 2025 National Academies Report, it is apparent that all four levels are needed to produce a more robust level of high-quality evidence in team science research.

**Table 1:** Proposed domains for evidence for team science.

Domain level	Domain	Description	Potential research examples
1	Evidence of constructs	Construct development and validation	Psychological safety in science teams; collective orientation of team members in nascent scientific teams
2	Evidence of causal relations	Statistical significance and/or observational concordance between constructs and outcome criteria	Use of transformational leadership to increase scientific team innovation; use of team mentoring to predict and produce career success
3	Evidence of interventional Effectiveness	Experimental and quasi-experimental evaluation of construct application; changes in individual criterion and team processes, development, and work outcomes	Use of team building to develop scientific team vision, roles, and responsibilities to measure effects upon timelines and milestones; training scientific team members in team review, reflection, and team development planning to determine effects upon number of publications over a three-year period
4	Evidence of system-wide impact	Impact analysis of aggregated findings across large agency and funding source using criteria established at agency level	Use of required ethics training upon number of long-term inter-institutional collaborations; implementation of standardized reporting of boundary spanning initiatives and number of new institutions and researchers within a research network

Notably, each of these domains reflects the positivist orientation of SciTS. Forms of ethnographic and other in-depth qualitative research favored by interpretive disciplines are not prominent. By expanding the view of what counts as high-quality evidence to include knowledge from interpretive fields, SciTS stands to gain additional ways of generating knowledge to expand its influence.

## Interpretive ethnographic and in-depth qualitative research as high-quality evidence in SciTS

Approaches such as short-term observations, evaluation questionnaires, evidence synthesis, consolidating expert consensus, and performing RCTs can only provide limited insights to understand what really works within scientific teams. While these approaches offer useful knowledge, in-depth ethnographic and qualitative approaches from Science and Technology Studies (STS), anthropology, sociology, and allied disciplines can offer a different kind of long-term and deeply contextualized analysis of why particular team science interventions and practices work or fail (Forsythe, 2001). Notably, valuing qualitative and interpretive ways of knowing as high-quality evidence will require SciTS to recognize that standards of reproducibility and generalizability favored by positivist approaches are not generally part of rigorous design standards in these disciplines. However, these fields have other standards of rigor rooted in longstanding disciplinary norms and traditions (Dourish, 2006; Latour, 2005; Ribes, 2019). For example, it is possible to create knowledge from long-term observations that have applicability to other settings, without relying on positivist standards of generalizability. In fact, most qualitative and ethnographic research is premised on this idea. In qualitative research teams studying scientists, members of the qualitative team must work together to foster shared understanding of their objects of study, ensuring both internal validity within the research team and mastery of the disciplinary languages of the group being studied. This approach requires embeddedness within research ecosystems that facilitate shared understanding and knowledge-translation across disciplinary worlds. While one conducts qualitative research narrowly and in specific contexts, one must also think broadly about the potential applicability of the research to other settings (Marcus and Fischer, 1999). The strength in ethnographic and other in-depth qualitative methods for SciTS lies in these approaches' ability to support the creation of best practices through deep contextual knowledge that is attendant to power relations within groups, structural factors that affect group behavior, unstated cultural norms, and related factors that are only observable through embeddedness and close attention to scientists' language and actions (Martinez Tyson et al., 2025).

We thus suggest that rigorously designed in-depth qualitative research should be valued as high-quality evidence in SciTS. We propose three potential ways that incorporating these approaches into SciTS can support the field's overall goals and contribute to its evidence-base.

First, undertaking ethnographic or other in-depth qualitative studies of SciTS interventions or best practices that have been shown to be effective can improve SciTS and its impact. Best practices that emerge from quantitative studies, through replication, trialing, or other forms of validation, can be more robustly understood through paired qualitative inquiry. Indeed, only an embedded qualitative approach can contextualize best practices that are emergent from positivist studies focused on the optimization of approaches, because interpretive methods reveal the deep structural factors that cause certain interventions or practices to work or fail. Further, through contextualization, qualitative forms of evidence can help in the translation of best practices from one team type to another (e.g., from aerospace teams to science teams).

Second, we suggest that SciTS practitioners use their existing programs of research and evaluation to pursue more robustly mixed methods approaches to evaluating team science trainings, best practices, and interventions. This would involve incorporating experts from the interpretive qualitative social sciences into existing team science research groups. Further, pursuing this approach would involve co-constructing research questions and approaches in the to accommodate varied epistemic viewpoints (e.g., positivist vs. interpretive). For example, building a long-term interview and observational component into an RCT of a team science training intervention would need to accommodate the disciplinary norms of the trialist and the qualitative social scientist. In another vein, a traditional evaluator would need to invest more time and resources into their studies to enable long-term observations, rather than a smaller number of interviews. Many arrangements are possible to more rigorously pursue mixed methods research in SciTS, but all would involve elevating the value of qualitative and interpretive ways of knowing.

Third, there is intrinsic value in the kinds of knowledge created by long-term embedded qualitative studies of science teams, including studies that are not connected to interventions. There are rich traditions of such studies in STS and the sociology and anthropology of science and technology, which have played a role in reshaping science policy and practice (Rabinow and Bennett, 2012). When put toward the applied end of helping to understand and improve science teams, qualitative knowledges should be treated as high-quality evidence that contributes to the further development of SciTS. Studies in this vein can be developed around naturalistic observations of science teams or as part of an interventional undertaking to better understand the effectiveness of a training or set of best practices adopted by a team. Ethnography's focus on deep contextual knowledge and the method's overall appreciation for contingency and the unexpected make it well-suited to describe why certain teams' approaches succeed or fail—including within teams or organizations that may not have received team science trainings (Martinez Tyson et al., 2025). To support this research, long-term, standalone ethnographic studies of science teams should become a priority for SciTS.

## Conclusion

It is time for SciTS to build upon the positivist paradigm in evaluating the quality of evidence and to recognize the value of interpretive approaches. Ethnographic and other in-depth qualitative studies are also potential sources of high-quality evidence for SciTS and can enhance the field's reach. Interpretive social studies of sciences have their own standards of rigor that can inform the applied aims of SciTS.

The payoffs for SciTS in investing in interpretive research are evident, insofar as these areas would generate knowledge not currently part of SciTS. However, there are also payoffs for fields within the interpretive social studies of science. Building alliances with SciTS offers opportunities for scholars engaged in interpretive social studies of science to translate their skills into new applied domains to understand, shape, and improve the conduct of science as well as the values of scientists and science teams.

The largest challenge facing SciTS practitioners and interpretive scholars working in social studies of science is the potential incommensurability of the epistemic paradigms that exist across fields. The positivist worldview, which posits stable hierarchies of evidence and promotes concepts such as generalizability and reproducibility, is arguably at fundamental odds with interpretivist worldviews where analyses of deep context, structure, culture, and change-over-time are paramount and where reproducibility and generalizability are not goals. In future efforts to bridge across disciplinary divides to bring in-depth qualitative, ethnographic, and interpretive ways of knowing into SciTS, it will be paramount to overcome these differences and to produce new transdisciplinary styles of innovation.

## Data Availability

The original contributions presented in the study are included in the article/supplementary material, further inquiries can be directed to the corresponding author.

## References

[B1] Bisbey T. M. Wooten K. C. Salazar Campo M. Lant T. K. Salas E. (2021). Implementing an evidence-based competency model for science team training and evaluation: teamMAPPS. J. Clin. Transl. Sci. 5:e142. doi: 10.1017/cts.2021.795 34422322 PMC8358845

[B2] Bluhm R. (2016). “*The hierarchy of evidence, meta-* analysis, and systematic review,” in The Routledge Companion to Philosophy of Medicine, Eds., M. Solomon, J. R. Simon, and H. Kincaid (New York, NY: Routledge), 199–209.

[B3] Bollen K. Cacioppo J. T. Kaplan R. M. Krosnick J. A. Olds J. L. (2015). Social, Behavioral, and Economic Sciences Perspectives on Robust and Reliable Science: Report of the Subcommittee on Replicability in Science Advisory Committee to the National Science Foundation Directorate for Social, Behavioral, and Economic Sciences. National Science Foundation.Available online at: https://rcra.emory.edu/_includes/documents/sections/oric/8-social-behavioral-and-economic-sciences-perspectives-on-robust-and-reliable-science.pdf (Accessed November 1, 2025).

[B4] Börner K. Contractor N. Falk-Krzesinski H. J. Fiore S. M. Hall K. L. Keyton J. . (2010). A multi-level systems perspective for the science of team science. Sci. Transl. Med. 2:1399. doi: 10.1126/scitranslmed.3001399 PMC3527819 20844283

[B5] Bozeman B. Boardman C. (2014). Research Collaboration and Team Science: A State-of-the-Art Review and agenda. Cham: Springer. doi: 10.1007/978-3-319-06468-0

[B6] Brasier A. R. Burnside E. S. Rolland B. (2023). Competencies supporting high-performance translational teams: a review of the sciTS evidence base. J. Clin. Transl. Sci. 7:e62. doi: 10.1017/cts.2023.17 37008597 PMC10052558

[B7] Chang M. (2017). what constitutes science and scientific evidence: roles of null hypothesis testing. Educ. Psychol. Meas. 77, 475–488. doi: 10.1177/0013164416667978 29795924 PMC5965552

[B8] Dourish P. (2006). “Implications for design,” in Proceedings of the SIGCHI Conference on Human Factors in Computing Systems—CHI'06, 541. New York, NY: ACM.

[B9] Evans D. (2003). Hierarchy of evidence: a framework for ranking evidence evaluating healthcare interventions. J. Clin. Nurs. 12, 77–84. doi: 10.1046/j.1365-2702.2003.00662.x 12519253

[B10] Falk-Krzesinski H. J. Börner K. Contractor N. Fiore S. M. Hall K. L. Keyton J. . (2010). Advancing the science of team science. Clin. Transl. Sci. 3, 263–266. doi: 10.1111/j.1752-8062.2010.00223.x 20973925 PMC2965626

[B11] Falk-Krzesinski H. J. Contractor N. Fiore S. M. Hall K. L. Kane C. Keyton J. . (2011). Mapping a research agenda for the science of team science. Res. Eval. 20, 143–156. doi: 10.3152/095820211X12941371876580 PMC3513779 23223093

[B12] Felt U. (ed). (2017). The Handbook of Science and Technology Studies Edn., 4th ed. Cambridge, MA: The MIT Press.

[B13] Fiore S. M. (2008). Interdisciplinarity as teamwork: how the science of teams can inform team science. Small Gr. Res. 39, 251–277. doi: 10.1177/1046496408317797

[B14] Forsythe D. (2001). Studying Those Who Study Us: An Anthropologist in The World Of Artificial Intelligence, ed.D. J. Hess Stanford, CA: Stanford University Press. doi: 10.1515/9781503619371

[B15] Goodman S. N. Royall R. (1988). Evidence and scientific research. Am. J. Public Health 78, 1568–1574. doi: 10.2105/AJPH.78.12.1568 3189634 PMC1349737

[B16] Hall K. L. Feng A. X. Moser R. P. Stokols D. Taylor B. K. (2008). Moving the science of team science forward. Am. J. Prev. Med. 35, S243–S249. doi: 10.1016/j.amepre.2008.05.007 18619406 PMC3321548

[B17] Hall K. L. Stokols D. Stipelman B. A. Vogel A. L. Feng A. Masimore B. . (2012). Assessing the value of team science. Am. J. Prev. Med. 42, 157–163. doi: 10.1016/j.amepre.2011.10.011 22261212 PMC3586819

[B18] Hall K. L. Vogel A. L. Croyle R. T. (2019). Strategies for Team Science Success: Handbook of Evidence-Based Principles for Cross-Disciplinary Science and Practical Lessons Learned from Health Researchers. Cham: Springer. doi: 10.1007/978-3-030-20992-6

[B19] Hall K. L. Vogel A. L. Huang G. C. Serrano K. J. Rice E. L. Tsakraklides S. P. . (2018). The science of team science: a review of the empirical evidence and research gaps on collaboration in science. Am. Psychol. 73, 532–548. doi: 10.1037/amp0000319 29792466

[B20] Hawk L. W. Murphy T. F. Hartmann K. E. Burnett A. Maguin E. (2024). A randomized controlled trial of a team science intervention to enhance collaboration readiness and behavior among early career scholars in the clinical and translational science award network. Am. Psychol. 8:e6. doi: 10.1017/cts.2023.692 PMC10877513 38384923

[B21] Kotarba J. A. (2014). Symbolic interaction and applied social research: a focus on translational science: basic and applied interactionist research. Symb. Interact. 37, 412–425. doi: 10.1002/symb.111 25221375 PMC4159952

[B22] Kotarba J. A. Molldrem S. Smith E. Spratt H. Bhavnani S. K. Farroni J. . (2023). Exploring team dynamics during the development of a multi-institutional cross-disciplinary translational team: implications for potential best practices. J. Clin. Transl. Sci. 7, 1–21. Cambridge Core. doi: 10.1017/cts.2023.640 PMC10643934 38028346

[B23] Kozlowski S. W. J. (2018). Enhancing the effectiveness of work groups and teams: a reflection. Perspect. Psychol. Sci. 13, 205–212. doi: 10.1177/1745691617697078 29232536

[B24] Latour B. (1987). Science in Action: How to Follow Scientists and Engineers Through Society. Cambridge, MA: Harvard University Press.

[B25] Latour B. (2005). Reassembling the Social: An Introduction to Actor-Network-Theory. Oxford: Oxford University Press.

[B26] Latour B. Woolgar S. (1979). Laboratory Life: The Construction of Scientific Facts. London: SAGE Publications.

[B27] LePine J. A. Piccolo R. F. Jackson C. L. Mathieu J. E. Saul J. R. (2008). A meta-analysis of teamwork processes: tests of a multidimensional model and relationships with team effectiveness criteria. Pers. Psychol. 61, 273–307. doi: 10.1111/j.1744-6570.2008.00114.x

[B28] Lerner D. Palm M. E. Concannon T. W. (Eds). (2022). Broadly Engaged Team Science in Clinical and Translational Research. Cham: Springer International Publishing. doi: 10.1007/978-3-030-83028-1

[B29] Little M. M. St Hill C. A. Ware K. B. Swanoski M. T. Chapman S. A. Lutfiyya M. N. . (2017). Team science as interprofessional collaborative research practice: a systematic review of the science of team science literature. **J. Investig. Med**. 65, 15–22. doi: 10.1136/jim-2016-000216 PMC5284346 27619555

[B30] Lotrecchiano G. R. Schwartz L. Falk-Krzesinski H. J. (2021). Measuring motivation for team science collaboration in health teams. J. Clin. Transl. Sci. 5:e84. doi: 10.1017/cts.2020.567 PMC8111607 34007467

[B31] Marcus G. E. Fischer M. M. J. (1999). Anthropology as cultural critique: An experimental moment in the human sciences Edn., 2nd ed. Chicago, IL: University of Chicago Press.

[B32] Martinez Tyson D. Dahlberg B. Higashi R. T. (2025). Bridging two worlds: challenges and opportunities for anthropologists working in team science. Pract. Anthropol. 47, 244–253. doi: 10.1080/08884552.2025.2551588

[B33] Mathieu J. Maynard M. T. Rapp T. Gilson L. (2008). Team effectiveness 1997-2007: a review of recent advancements and a glimpse into the future. J. Manage. 34, 410–476. doi: 10.1177/0149206308316061

[B34] Mathieu J. E. Gallagher P. T. Domingo M. A. Klock E. A. (2019). Embracing complexity: reviewing the past decade of team effectiveness research. Annu. Rev. Organ. Psychol. Organ. Behav. 6, 17–46. doi: 10.1146/annurev-orgpsych-012218-015106

[B35] McGuier E. A. Wright J. D. Flett G. Rothenberger S. D. Salas E. Kolko D. J. (2025). Team training in the real world: a cluster-randomized hybrid effectiveness-implementation trial of teamtracs in rural children's advocacy centers. J. Clin. Transl. Sci. 9:e275. doi: 10.1017/cts.2025.10203 41523639 PMC12779490

[B36] Mendell A. M. Knerich V. Ranwala D. (Dayan), Jones, C. T. Piechowski P. Striley C. W. McCormack W. T. . (2024). Team science competencies across the career life course for translational science teams. J. Clin. Transl. Sci. 8, 1–24. doi: 10.1017/cts.2024.494 PMC11626591 39655023

[B37] Morrison M. (2006). Applying science and applied science: what's the difference? Int. Stud. Philos. Sci. 20, 81–91. doi: 10.1080/02698590600641057

[B38] National Academies of Sciences Engineering and Medicine. (2015). Enhancing the Effectiveness of Team Science (p. 19007). Washington, DC: National Academies Press. 26247083

[B39] National Academies of Sciences Engineering, and Medicine (ed).. (2025). The Science and Practice of Team Science, Edn., 1st ed. Washington, DC: National Academies Press. 40554653

[B40] Rabinow P. Bennett G. (2012). Designing Human Practices: An Experiment With Synthetic Biology. Chicago, IL: The University of Chicago Press.

[B41] Rhodes T. Lancaster K. (2019). Evidence-making interventions in health: a conceptual framing. Soc. Sci. Med. 238:112488. doi: 10.1016/j.socscimed.2019.112488 31422173

[B42] Ribes D. (2019). STS, meet data science, once again. Sci. Technol. Hum. Values 44, 514–539. doi: 10.1177/0162243918798899

[B43] Ribes D. Baker K. (2007). “Modes of Social Science Engagement in Community Infrastructure Design,” in Communities and Technologies 2007, eds. C. Steinfield, B. T. Pentland, M. Ackerman, and N. Contractor (London: Springer London), 107–130. doi: 10.1007/978-1-84628-905-7_6

[B44] Rolland B. Cross J. E. Hohl S. D. Johnson L. J. Wooten K. Brasier A. R. (2021a). Introduction to the themed issue on the design, development, evaluation, and dissemination of team science interventions in clinical and translational research. J. Clin. Transl. Sci. 5:e202. doi: 10.1017/cts.2021.870 35047214 PMC8727717

[B45] Rolland B. Hohl S. D. Johnson L. J. (2021b). Enhancing translational team effectiveness: the wisconsin interventions in team science framework for translating empirically informed strategies into evidence-based interventions. J. Clin. Transl. Sci. 5:e158. doi: 10.1017/cts.2021.825 34527297 PMC8427550

[B46] Shaw J. (2022). Revisiting the basic/applied science distinction: the significance of urgent science for science funding policy. J. Gen. Philos. Sci. 53, 477–499. doi: 10.1007/s10838-021-09575-1 35106028 PMC8796194

[B47] Stokols D. Hall K. L. Taylor B. K. Moser R. P. (2008). The science of team science. Am. J. Prev. Med. 35, S77–S89. doi: 10.1016/j.amepre.2008.05.002 18619407

[B48] Taper M. L. Lele S. (eds). (2004). The Nature of Scientific Evidence: Statistical, Philosophical, and Empirical Considerations. Chicago, IL: University of Chicago Press. doi: 10.7208/chicago/9780226789583.001.0001

[B49] Vogel A. L. Knebel A. R. Faupel-Badger J. M. Portilla L. M. Simeonov A. (2021). A systems approach to enable effective team science from the internal research program of the national center for advancing translational sciences. J. Clin. Transl. Sci. 5:e163. doi: 10.1017/cts.2021.811 34527302 PMC8427549

[B50] Vogel A. L. Stipelman B. A. Hall K. L. Nebeling L. Stokols D. Spruijt-Metz D. (2014). Pioneering the transdisciplinary team science approach: lessons learned from national cancer institute grantees. J. Transl. Med. Epidemiol. 2:1027. 25554748 PMC4280018

[B51] Wooten K. C. Rose R. M. Ostir G. V. Calhoun W. J. Ameredes B. T. Brasier A. R. (2014). Assessing and evaluating multidisciplinary translational teams: a mixed methods approach. Eval. Health Prof. 37, 33–49. doi: 10.1177/0163278713504433 24064432 PMC4180502

[B52] Yaghmaie A. (2017). How to characterise pure and applied science. Int. Stud. Philos. Sci. 31, 133–149. doi: 10.1080/02698595.2018.1424763

